# Full vaccination coverage with valid doses among the 2017 and 2018 live birth cohort in the Southeast region of Brazil

**DOI:** 10.1590/S2237-96222024v33e2024433.especial2.en

**Published:** 2024-12-16

**Authors:** Ana Paula França, Rita Barradas Barata, Ione Aquemi Guibu, José Cássio de Moraes, ICV 2020 Group, Adriana Ilha da Silva, Alberto Novaes Ramos, Ana Paula França, Andrea de Nazaré Marvão Oliveira, Antonio Fernando Boing, Carla Magda Allan Santos Domingues, Consuelo Silva de Oliveira, Ethel Leonor Noia Maciel, Ione Aquemi Guibu, Isabelle Ribeiro Barbosa Mirabal, Jaqueline Caracas Barbosa, Jaqueline Costa Lima, José Cássio de Moraes, Karin Regina Luhm, Karlla Antonieta Amorim Caetano, Luisa Helena de Oliveira Lima, Maria Bernadete de Cerqueira Antunes, Maria da Gloria Teixeira, Maria Denise de Castro Teixeira, Maria Fernanda de Sousa Oliveira Borges, Rejane Christine de Sousa Queiroz, Ricardo Queiroz Gurgel, Rita Barradas Barata, Roberta Nogueira Calandrini de Azevedo, Sandra Maria do Valle Leone de Oliveira, Sheila Araújo Teles, Silvana Granado Nogueira da Gama, Sotero Serrate Mengue, Taynãna César Simões, Valdir Nascimento, Wildo Navegantes de Araújo

**Affiliations:** 1 Faculdade de Ciências Médicas da Santa Casa de São Paulo, Departamento de Saúde Coletiva, São Paulo, SP, Brazil; Universidade Federal do Espírito Santo, Vitória, ES, Brazil; Universidade Federal do Ceará, Departamento de Saúde Comunitária, Fortaleza, CE, Brazil; Faculdade Ciências Médicas Santa Casa de São Paulo, São Paulo, SP, Brazil; Secretaria de Estado da Saúde do Amapá, Macapá, AP, Brazil; Universidade Federal de Santa Catarina, SC, Brazil; Organização Pan-Americana da Saúde, Brasília, DF, Brazil; Instituto Evandro Chagas, Belém, PA, Brazil; Faculdade de Ciências Médicas Santa Casa de São Paulo, Departamento de Saúde Coletiva, São Paulo, SP, Brazil; Universidade Federal de Mato Grosso, Cuiabá, MT, Brazil; Universidade Federal do Paraná, Curitiba, PR, Brazil; Universidade Federal de Goiás, Goiânia, GO, Brazil; Universidade Federal do Piauí, Teresina, PI, Brazil; Universidade de Pernambuco, Faculdade de Ciências Médicas, Recife, PE, Brazil; Instituto de Saúde Coletiva, Universidade Federal da Bahia, Salvador, BA, Brazil; Secretaria de Estado da Saúde de Alagoas, Maceió, AL, Brazil; Universidade Federal do Acre, Rio Branco, AC, Brazil; Universidade Federal do Maranhão, Departamento de Saúde Pública, São Luís, MA, Brazil; Universidade Federal de Sergipe, Aracaju, SE, Brazil; Secretaria Municipal de Saúde, Boa Vista, RR, Brazil; Fundação Oswaldo Cruz, Mato Grosso do Sul, Campo Grande, MS, Brazil; Fundação Oswaldo Cruz, Escola Nacional de Saúde Pública Sergio Arouca, Rio de Janeiro, RJ, Brazil; Universidade Federal do Rio Grande do Sul, Porto Alegre, RS, Brazil; Fundação Oswaldo Cruz, Instituto de Pesquisa René Rachou, Belo Horizonte, MG, Brazil; Secretaria de Desenvolvimento Ambiental de Rondônia, Porto Velho, RO, Brazil; Universidade de Brasília, Brasília, DF, Brazil

**Keywords:** Cobertura de Vacunación, Salud Infantil, Factores Socioeconómicos, Encuestas Epidemiológicas, Vaccination Coverage, Child Health, Socioeconomic Factors, Health Surveys

## Abstract

**Objective:**

To analyze factors associated with full vaccination coverage with valid doses, in children from four state capitals and three other cities in Southeast Brazil.

**Method:**

Analysis of a population survey conducted in 2020-2021, with a sample stratified according to socioeconomic levels of children born in 2017-2018, with data collected through photographic records of their vaccination cards. Odds ratios (OR) and 95% confidence intervals (95%CI) for full vaccination coverage were estimated based on the characteristics of the family, mother and child.

**Results:**

Among 8703 children, lowest coverage occurred in strata A and B (OR=0.39; 95%CI 0.23;0.67 and OR=0.38; 95%CI 0.25;0.58); in consumption level A/B (OR=0.38; 95CI% 0.28;0.52); among those with income >BRL8000/month (OR=0.23; 95%CI 0.12;0.42); in children of mothers with higher education (OR=0.47; 95%CI 0.32;0.71); in children not vaccinated exclusively in the public service (OR=0.37; 95%CI 0.26;0.51) and in children with a vaccination delay of up to 6 months (OR=0.28; 95%CI 0.22;0.37).

**Conclusion:**

Coverage did not reach the targets for controlling vaccine-preventable diseases and was negatively associated with higher socioeconomic status.

## Study contributions

**Main results:**

Vaccination coverage for the full schedule with valid doses was very low, putting the control of vaccine-preventable diseases at risk in the four state capitals and three other cities in the interior region of Southeast Brazil.

**Implications for services:**

Brazilian National Health System managers and workers need to know the factors associated with low vaccination coverage and increased risk of abandoning the schedule, access problems and National Immunization Program difficulties.

**Perspectives:**

Understanding determinants of low vaccination coverage provides support for the discussion and design of effective public policies to address barriers and qualify health services for vaccination.

## RESUMEN

**Objetivo:**

Analizar factores asociados a la cobertura completa de vacunación con dosis válidas, en niños de las capitales y de otras tres ciudades de la región sureste del Brasil.

**Método:**

Análisis de una encuesta poblacional realizada en 2020-2021, con una muestra estratificada según niveles socioeconómicos de los niños nacidos en 2017-2018, con datos recolectados a través de registros fotográficos de sus cartillas de vacunación. Se estimaron los odds ratios (OR) y los intervalos de confianza del 95% (IC95%) para la cobertura completa de vacunación en función de las características de la familia, la madre y el niño.

**Resultados:**

En 8703 niños hubo menor cobertura en los estratos A y B (OR=0,39; IC95% 0,23;0,67 y OR=0,38; IC95% 0,25;0,58); con nivel de consumo A/B (OR=0,38; IC95% 0,28;0,52); con renta >BRL8.000/mes (OR=0,23; IC95% 0,12;0,42); en hijos de madres con educación superior (OR=0,47; IC95% 0,32;0,71); en niños no vacunados exclusivamente en servicio público (OR=0,37; IC95% 0,26;0,51) y en niños con retraso vacunal hasta 6 meses (OR=0,28; IC95% 0,22;0,37).

**Conclusión:**

La cobertura no alcanzó metas de control de enfermedades inmunoprevenibles y se asoció negativamente con mayor nivel socioeconómico.

## INTRODUCTION

The Brazilian National Immunization Program (*Programa Nacional de Imunizações* - PNI) established in 1973^1^ is recognized as one of the most complete in the world and for having achieved, for years, high vaccination coverage due to the universal and free nature of the Brazilian National Health System (*Sistema* Único *de Saúde* – SUS).^2^

However, from 2012 onwards, a decline in vaccination coverage was detected, worsening in 2016 and, even more so, due to the COVID-19 pandemic.^3^ Data from the National Immunization Program Information System (SI-PNI) showed that, between 2018 and 2019, the number of administered doses of seven vaccines decreased in all Brazilian regions: monovalent rotavirus; pneumococcal conjugate; hepatitis B; BCG; inactivated and oral poliovirus; diphtheria, tetanus and pertussis (DTP); and DTP, *Haemophilus influenza* type B and hepatitis B. Only coverage of varicella and meningococcal serogroup C vaccines increased.^4^

Despite the pioneering spirit in the implementation and development of PNI immunization actions, the Southeast, as well as other regions of Brazil, have recorded a drop in vaccination coverage, with levels below the national schedule targets.^5^ In 2020, doses administered to children up to 10 years old fell 9.4% compared to 2019, with the Southeast being one of the most affected regions (-12.8%).^6^ Poliovirus coverage, according to SI-PNI data, decreased by almost 30% between 2011 and 2021, this being a trend found in 24 of the 27 Brazilian Federative Units (i.e. 26 states and the Federal District).^7^

Considering average coverage of vaccines on the childhood schedule, per Federative Unit as per the SI-PNI, a worrying decline was found in the period from 2016 to 2020 in all four Southeast region states: 24.0% in Espírito Santo, 22.5% in Minas Gerais, 50.3% in Rio de Janeiro and 28.1% in São Paulo.^8^

It should be noted that there are indications of discrepancies in vaccination coverage according to SI-PNI data when compared to survey data, which indicate possible failures in recording administered doses on the information system (SI-PNI) and in estimating the target population.^9^

Household surveys produce more precise estimates, enabling estimates of children with a full vaccination schedule and the proportion of susceptible children, as well as enabling understanding of the socioeconomic determinants of the heterogeneous distribution of childhood vaccination coverage and factors related to equity of access to the PNI.^10^

The objective of this article was to analyze factors associated with full vaccination coverage with valid doses, in children in the four state capitals and in three other cities in the Southeast region of Brazil.

## METHODS

This is an analysis of the National Vaccination Coverage Survey, a population-based study, conducted from 2020 to 2022 regarding children born in 2017 and 2018, living in the urban area of the 26 Brazilian state capitals, Federal District and in 12 interior region cities with more than 100,000 inhabitants. 

The present study verified adherence to the PNI vaccination schedule up to 24 months old among children born in the four state capitals of the Southeast region (Belo Horizonte, MG; Vitória, ES; Rio de Janeiro, RJ and São Paulo, SP) and in three other cities in the region (Sete Lagoas, MG; Petrópolis, RJ and Campinas, SP).

In the first stage, the census tracts were classified into four socioeconomic strata (A, B, C and D, with stratum A being the one with the best socioeconomic status, and stratum D with the poorest) in each of the 39 municipalities covered by the national survey. The strata classification cut-off points were different between the municipalities, being defined according to data on the nominal income of the heads of family, the percentage of heads of family with income above 20 minimum wages and the percentage of literate heads of family, as per the 2010 Demographic Census.^11^

The sample size calculation considered vaccination coverage of 70%, design effect of 1.4 and a 95% confidence interval, which resulted in 452 children in each survey. The number of surveys in each municipality was defined based on its population, resulting in 452 children in Sete Lagoas and Petrópolis, 904 in Vitória and 1,808 children in Belo Horizonte, Rio de Janeiro, São Paulo and Campinas.^11^

The census tracts were grouped together by proximity and expected number of children, so that each cluster contained three times the number of children to be included in the sample in order to compensate for possible address errors and other losses. The clusters were systematically selected at random.^11^

The vaccine administration dates were obtained by photographing the children’s vaccination cards. The socioeconomic characteristics of families, mothers and children and questions about vaccination hesitancy were obtained through interviews using a structured questionnaire administered by a trained interviewer.

The doses of different vaccines aimed at preventing the same diseases were added up (for example: MMR [measles, mumps and rubella] and MMRV [measles, mumps, rubella and varicella]), in order to correctly calculate coverage considering the vaccines administered by both public and private services.^12^

Taking the difference between the dates recorded on the vaccination cards and the children’s date of birth, the doses were able to be classified as valid when applied 15 days before the date set by the PNI, respecting the minimum interval recommended for each dose.^12^

The outcome was full vaccination coverage with valid doses, defined as administration of all doses and boosters of vaccines on the official PNI schedule up to 24 months old: Bacillus Calmette-Guérin (BCG); hepatitis B (HepB); diphtheria, tetanus, pertussis, *hemophilus influenza* B, hepatitis B (DTcP-Hib-HepB:) – first + second + third doses; inactivated poliovirus 1, 2 and 3 (IVP: first + second + third doses); rotavirus (RV1: first and second doses); meningococcal serogroup C (MENC: first and second doses + booster); pneumococcal conjugate (PCV10: first and second doses); measles, mumps and rubella (MMR: first and second doses + booster); hepatitis A (HepA: first dose); varicella (VAR: single dose); attenuated oral poliovirus 1 and 3 (bOPV: booster); diphtheria, tetanus and pertussis (DTP: booster). Yellow fever vaccine was excluded from the calculation, as it was not part of the routine schedule in some municipalities.^12^

The covariables included the following blocks.

- Family characteristics: socioeconomic stratum (A, B, C and D); family consumption level (A/B and C/D), defined according to the criteria used by the Brazilian Association of Survey Companies;^13^ monthly family income (≤BRL 1000, BRL 1001-3000, BRL 3001-8000, ≥BRL 8001); household crowding (no, yes=≥3 persons per bedroom); and federal income transfer program beneficiary (yes, no).

- Maternal characteristics: schooling (no schooling or incomplete elementary education, complete elementary education or incomplete high school education, complete high school education or incomplete higher education, complete higher education); paid work in the last month (yes, no); age group (<20 years, 20-34 years, ≥35 years); has a partner (yes, no); and number of children (1, 2 and ≥3).

- Child characteristics: sex (male, female); race/skin color (White, Black or mixed race); attends daycare or school (yes, no); birth order (first, second, third or above); difficulties accessing vaccination service (yes, no); any delayed vaccination up to 6 months old (yes, no); and whether the child received on the same date the vaccines recommended at 4 months (yes, no).

The coverage cascade for vaccines on the PNI schedule for the first 24 months of life started with BCG, calculated taking the number of children vaccinated with valid doses of that vaccine as the numerator and the number of children in the sample as the denominator. Subsequently, the numerator was the number of children who received valid doses of a given vaccine and the denominator was the number of valid doses of the vaccine immediately administered before it. Therefore, in order to calculate HepB coverage at birth, the number of children who received valid HepB doses was divided by the number of children vaccinated with valid BCG doses.

Vaccination coverage estimates and their respective 95% confidence intervals were calculated using the Stata® (version 17) survey analysis module, considering the sample weights and the study design. Pearson’s chi-squared test was used to test for associations between the outcome and covariables (p-value<0.05). Using logistic regression, crude odds ratios (OR) adjusted for age and maternal education were estimated, with their respective confidence intervals. 

The survey was approved by the Research Ethics Committee of the *Instituto de Saúde Coletiva da Universidade Federal da Bahia*, as per Opinion No. 3.366.818, on June 4, 2019, and Certificate of Submission for Ethical Appraisal (*Certificado de Apresentação de Apreciação* Ética – CAAE) No. 4306919.5.0000.5030; and by the Research Ethics Committee of the *Irmandade da Santa Casa de São Paulo*, as per Opinion No. 4.380.019, on November 4, 2020, and CAAE No. 39412020.0.0000.5479. 

## RESULTS

A total of 8,703 children born between 2017 and 2018 took part in this study, in Belo Horizonte (n=1,863, 103.0% of predicted), Vitória (n=788, 87.2% of predicted), Rio de Janeiro (n=1,820, 100.7% of predicted), São Paulo (n=1,539, 85.1% of predicted), Sete Lagoas (n=451, 99.6% of predicted), Petrópolis (n=468, 103.5% of predicted ) and Campinas (n=1,774, 98.1% of predicted).

In the Southeast region, the proportion of children with full vaccination coverage with valid doses at 24 months was estimated at 41.3% (95%CI 37.9;44.9) for the capitals and 47.1% (95% CI 40.8;53.4) for the three cities in the interior. The city with the highest vaccination coverage was Sete Lagoas (61.6%; 95%CI 53.6;69.1), and those with the lowest coverage were Vitória (34.4%; 95%CI 23.3;47.2) and Rio de Janeiro (34.6%; 95%CI 29.9;39.6). In the other cities, vaccination coverage was 43.3% (95%CI 35.9;51.0) in Campinas, 44.2% (95%CI 39.3;49.3) in São Paulo, 46.2% (95%CI 40.2;52.3) in Belo Horizonte and 50.3% (95%CI 34.4;66.1) in Petrópolis (Table 1). 

**Table 1:** Full vaccination coverage with valid doses (%) and 95% confidence intervals (95%CI) according to family characteristics, National Vaccination Coverage Survey, Belo Horizonte, Sete Lagoas, Vitória, Rio de Janeiro, Petrópolis, São Paulo, Campinas and Brazil, 2020 (n=8,703)

Characteristics	**Minas Gerais**		Espírito Santo	**Rio de Janeiro**		São Paulo		Southeast	
	**Belo Horizonte (n=1,863)**	**Sete Lagoas (n=451)**	**Vitória (n=788)**	**Rio de Janeiro (n=1,820)**	**Petrópolis (n=468)**	**São Paulo (n=1,539)**	**Campinas (n=1,774)**	**Capitals (n=6,010)s**	**Interior (n=2,693)**
**Socioeconomic stratum**									
A	28.6 (21,3;37.1)	51.4 (38.2;64.4)	3.5 (0.9;12.4)	18.1 (6.5;41.5)	19.7 (4.5;55.9)	32.0 (19.0;48.5)	12.0 (5.7;23.6)	24.1 (15.2;35.9)	16.6 (9.3;28.0)
B	28.1 (19.4;39.0)	55.0 (42.6;66.9)	39.4 (28.5;51.5)	16.6 (8.9;28.8)	56.4 (47.4;65.1)	20.0 (11.3;32.8)	47.5 (37.2;58.0)	20.0 (14.0;27.8)	49.5 (41.2;57.8)
C	43.6 (35.3;52.2)	54.9 (41.9;67.3)	34.0 (27.4;41.3)	42.8 (34.9;51.1)	70.4 (53.7;83.0)	28.8 (20.7;38.5)	55.3 (49.0;61.5)	35.3 (29.5;41.7)	58.0 (51.8;64.0)
D	51.5 (41.9;61.0)	69.3 (55.7;80.2)	60.1 (51.9;67.7)	38.0 (31.8;44.6)	56.6 (48.3;64.7)	51.6 (45.8;57.3)	56.7 (49.4;63.8)	47.7 (43.4;52.1)	59.1 (53.6;64.5)
**Family consumption level**									
A/B	38.0 (28.6;48.4)	53.2 (33.8;71.7)	19.2 (10.1;33.3)	20.2 (13.7;28.9)	13.5 (3.4;40.5)	24.6 (18.5;31.9)	26.7 (16.7;39.8)	24.3 (20.0;29.3)	27.9 (18.5;39.7)
C/D	49.3 (41.4;57.3)	63.6 (54.5;71.9)	55.8 (48.9;62.5)	39.9 (34.0;46.1)	55.7 (43.6;67.2)	52.2 (46.6;57.7)	57.1 (52.3;61.7)	48.3 (44.3;52.4)	57.9 (53.5;62.1)
**Monthly family income (BRL)**									
≤1000	57.5 (48.9;65.7)	52.3 (36.6;67.6)	64.5 (52.4;74.9)	38.6 (29.9;48.2)	45.3 (37.0;53.8)	53.5 (45.7;61.1)	55.0 (45.3;64.3)	49.8 (44.1;55.4)	52.1 (45.4;58.7)
1001-3000	49.9 (35.7;64.2)	66.2 (57.1;74.3)	52.8 (40.7;64.5)	41.7 (33.6;50.1)	55.3 (36.8;72.4)	53.8 (44.0;63.4)	56.1 (49.6;62.4)	50.0 (43.5;56.5)	57.6 (51.2;63.7)
3001-8000	41.4 (35.0;48.0)	69.0 (47.3;84.6)	30.7 (21.6;41.7)	40.6 (31.2;50.7)	46.1 (18.6;76.2)	39.8 (28.8;51.8)	46.4 (31.9;61.5)	40.1 (33.8;46.8)	48.1 (35.3;61.3)
≥8001	23.6 (10.2;45.5)	30.2 (8.7;66.1)	19.1 (6.5;44.5)	21.0 (11.5;35.3)	10.1 (1.5;45.4)	14.1 (5.1;33.1)	18.3 (9.0;33.7)	18.6 (11.9;28.0)	18.6 (9.6;33.1)
**Household crowding**									
Yes	61.7 (41.8;78.4)	66.2 (33.1;88.6)	62.8 (45.5;77.3)	27.5 (15.9;43.4)	35.3 (22.9;50.1)	37.4 (25.6;51.0)	45.1 (31.7;59.2)	34.8 (26.0;44.7)	45.4 (34.5;56.8)
No	45.5 (39.4;51.8)	61.4 (53.5;68.8)	32.9 (21.9;46.2)	36.2 (31.2;41.5)	51.3 (34.0;68.3)	45.1 (40.1;50.1)	43.2 (35.4;51.3)	42.3 (38.8;45.9)	47.2 (40.6;53.9)
**Income transfer program**									
Yes	51.6 (40.4;62.7)	56.2 (42.3;69.3)	65.9 (55.1;75.3)	49.7 (40.4;59.1)	44.6 (37.5;52.0)	53.5 (45.7;61.1)	49.7 (39.4;60.0)	52.5 (46.8;58.1)	49.6 (42.7;56.4)
No	44.9 (38.3;51.6)	63.3 (55.4;70.5)	28.0 (17.8;41.2)	31.1 (25.9;36.8)	52.3 (30.9;72.8)	41.5 (35.9;47.4)	42.1 (33.5;51.3)	38.4 (34.6;42.5)	46.6 (39.0;54.4)
**Total**	46.2 (40.2;52.3)	61.6 (53.6;69.1)	34.4 (23.3;47.2)	34.6 (29.9;39.6)	50.3 (34.4;66.1)	44.2 (39.3;49.3)	43.3 (35.9;51.0)	41.3 (37.9;44.9)	47.1 (40.8;53.4)

When assessing family characteristics, coverage was higher in socioeconomic stratum D, in Belo Horizonte, Vitória, São Paulo and Campinas, and in stratum C, in Rio de Janeiro and Petrópolis (p-value<0.05). In Sete Lagoas, no statistical difference was found according to strata. Lower vaccination coverage in the highest socioeconomic strata (A and B) was found in most cities (p-value<0.05), with the exception of Belo Horizonte and Sete Lagoas where there was no difference between the strata. Full vaccination coverage was lower in families with income ≥BRL 8001 in most cities (Belo Horizonte, Vitória, Rio de Janeiro, São Paulo and Campinas), as well as in all the capitals and interior region cities taken together (p-value<0. 05). Statistically higher coverage was found among children from families who were income transfer program beneficiaries in Vitória, Rio de Janeiro, São Paulo and in all the capitals taken together (Table 1).

Vaccination coverage with valid doses was lower (p-value<0.05) among children of mothers with higher schooling levels in Sete Lagoas, Vitória, São Paulo and Campinas and also in the capitals and interior region cities taken together. Children of mothers with paid work in the month preceding the interview also had lower vaccination coverage with valid doses (p-value<0.05), with the exception of Sete Lagoas and Campinas. Children of mothers aged 35 or over also had lower coverage in Vitória, São Paulo and in all the capitals and interior region cities taken together (Table 2).

**Table 2:** Full vaccination coverage with valid doses (%) and 95% confidence intervals (95%CI) according to maternal characteristics, National Vaccination Coverage Survey, Belo Horizonte, Sete Lagoas, Vitória, Rio de Janeiro, Petrópolis, São Paulo and Campinas, Brazil, 2020 (n=8,703)

Characteristics	**Minas Gerais**		Espírito Santo	**Rio de Janeiro**		São Paulo		Southeast	
	**Belo Horizonte (n=1,863)**	**Sete Lagoas (n=451)**	**Vitória (n=788)**	**Rio de Janeiro (n=1,820)**	**Petrópolis (n=468)**	**São Paulo (n=1,539)**	**Campinas (n=1,774)**	**Capitals (n=6,010)s**	**Interior (n=2,693)**
**Schooling**									
No schooling and incomplete elementary education	47.3 (31.9;63.2)	50.9 (29.5;72.0)	57.9 (39.8;74.0)	46.4 (30.0;63.7)	58.5 (36.3;77.7)	42.7 (33.1;53.0)	63.9 (46.0;78.6)	44.6 (36.4;53.2)	61.1 (48.3;72.5)
Complete elementary education	52.0 (43.4;60.5)	46.4 (28.7;65.0)	53.3 (41.4;64.9)	34.1 (22.9;47.3)	57.0 (45.0;68.3)	58.4 (46.6;69.4)	55.2 (43.2;66.6)	51.2 (42.5;59.9)	54.1 (46.0;61.9)
Complete high school education	50.5 (39.1;61.9)	70.9 (63.5;77.3)	61.4 (52.1;69.9)	37.6 (29.7;46.2)	60.0 (48.6;70.4)	51.4 (44.7;58.1)	59.5 (53.4;65.4)	47.9 (42.9;52.9)	61.8 (57.0;66.4)
Complete higher education	37.4 (30.3;45.0)	45.2 (30.8;60.4)	19.1 (10.5;32.4)	27.1 (20.9;34.2)	26.1 (7.0;62.6)	27.7 (21.3;35.1)	26.5 (17.2;38.3)	28.0 (23.8;32.8)	27.9 (19.2;38.7)
**Paid work in the last month**									
Yes	41.2 (35.5;47.2)	63.7 (51.3;74.5)	26.6 (14.9;42.7)	30.4 (25.1;36.2)	42.0 (24.2;62.1)	40.7 (34.3;47.5)	42.1 (31.1;54.0)	37.4 (33.1;42.0)	44.4 (35.4;53.9)
No	52.1 (43.1;60.9)	60.0 (49.3;69.8)	48.6 (41.0;56.2)	39.3 (32.6;46.5)	66.4 (52.2;78.1)	50.0 (43.4;56.5)	51.2 (40.1;62.1)	47.0 (42.4;51.6)	55.8 (47.7;63.6)
**Age group (years)**									
<20	50.6 (30.6;70.4)	76.7 (15.7;98.3)	38.8 (8.0;82.3)	44.6 (19.6;72.7)	35.6 (13.5;66.3)	48.8 (19.0;79.4)	91.2 (75.1;97.3)	47.6 (27.4;68.6)	78.0 (59.4;89.6)
20-34	46.3 (36.5;56.4)	61.9 (51.4;71.5)	48.7 (39.5;58.0)	38.0 (31.6;44.8)	60.4 (48.7;70.9)	50.4 (42.9;57.9)	51.1 (41.4;60.8)	46.1 (41.1;51.2)	54.8 (47.7;61.7)
≥35	46.0 (39.6;52.6)	61.1 (51.4;70.1)	25.0 (13.4;42.0)	30.8 (24.1;38.4)	36.4 (15.6;64.1)	38.2 (32.0;44.8)	36.8 (25.3;50.1)	36.4 (31.9;41.1)	39.8 (29.8;50.8)
**Partner**									
Yes	43.6 (36.0;51.4)	61.9 (52.7;70.3)	30.4 (19.2;44.5)	33.0 (27.9;38.5)	51.9 (30.9;72.2)	43.1 (37.1;49.2)	44.1 (35.5;53.0)	39.9 (35.8;44.2)	47.7 (40.2;55.4)
No	51.4 (41.8;60.8)	61.2 (51.6;69.9)	50.9 (40.9;61.0)	37.7 (29.9;46.3)	44.4 (32.3;57.3)	49.5 (40.6;58.4)	52.3 (39.8;64.6)	45.9 (40.3;51.7)	52.3 (44.9;59.7)
**Number of children**									
1	42.6 (33.8;51.8)	63.8 (52.8;73.5)	27.4 (17.1;40.9)	38.6 (30.2;47.6)	59.3 (40.2;76.0)	43.6 (35.8;51.8)	46.5 (35.8;57.6)	41.6 (36.2;47.1)	51.2 (42.7;59.7)
2	49.5 (41.1;58.0)	60.0 (47.8;71.1)	36.5 (22.1;53.9)	28.8 (21.7;37.1)	43.6 (18.3;72.7)	45.2 (37.5;53.0)	43.5 (35.3;52.1)	40.8 (35.5;46.3)	45.7 (37.3;54.3)
≥3	46.9 (40.5;53.4)	60.2 (46.4;72.6)	51.5 (40.4;62.5)	35.6 (26.4;46.0)	50.9 (39.5;62.2)	43.9 (36.2;51.9)	37.5 (22.3;55.6)	41.7 (36.0;47.6)	42.9 (30.1;56.6)
**Total**	46.2 (40.2;52.3)	61.6 (53.6;69.1)	34.4 (23.3;47.2)	34.6 (29.9;39.6)	50.3 (34.4;66.1)	44.2 (39.3;49.3)	43.3 (35.9;51.0)	41.3 (37.9;44.9)	47.1 (40.8;53.4)

The characteristics of the children had little impact on vaccination coverage with valid doses in the cities studied (Table 3). No statistically significant differences were found in relation to sex and race/skin color. Coverage among children who attended school and daycare was only higher in Campinas (p-value*-value*<0.05). 

**Table 3:** Full vaccination coverage with valid doses (%) and 95% confidence intervals (95%CI) according to child and service barrier characteristics, National Vaccination Coverage Survey, Belo Horizonte, Sete Lagoas, Vitória, Rio de Janeiro, Petrópolis, São Paulo and Campinas, Brazil, 2020 (n=8,703)

Characteristics	**Minas Gerais**		Espírito Santo	**Rio de Janeiro**		São Paulo		Southeast	
	**Belo Horizonte (n=1,863)**	**Sete Lagoas (n=451)**	**Vitória (n=788)**	**Rio de Janeiro (n=1,820)**	**Petrópolis (n=468)**	**São Paulo (n=1,539)**	**Campinas (n=1,774)**	**Capitals (n=6,010)s**	**Interior (n=2,693)**
**Sex**									
Male	42.8 (34.2;52.0)	64.4 (53.6;74.0)	38.5 (26.0;52.6)	35.8 (29.5;42.6)	39.8 (21.9;61.0)	45.2 (39.0;51.6)	43.5 (34.2;53.2)	42.0 (37.7;46.4)	45.6 (37.7;53.8)
Female	50.5 (44.9;56.0)	58.4 (49.1;67.2)	29.4 (18.7;43.1)	33.3 (27.2;40.1)	66.1 (51.5;78.2)	43.2 (36.9;49.8)	43.1 (33.4;53.4)	40.7 (36.2;45.3)	48.8 (41.0;56.7)
**Race/skin color**									
White	43.1 (36.0;50.5)	54.1 (39.5;68.1)	27.3 (18.7;37.9)	32.4 (26.4;39.1)	44.0 (23.6;66.6)	43.8 (38.2;49.6)	40.8 (30.7;51.7)	40.4 (36.2;44.7)	42.4 (33.6;51.6)
Black/mixed race	49.2 (42.0;56.4)	63.4 (53.1;72.6)	42.9 (26.7;60.8)	38.6 (30.9;47.0)	59.5 (49.7;68.5)	45.8 (37.7;54.1)	49.8 (37.5;62.0)	43.7 (38.6;48.9)	55.0 (46.9;62.8)
**Attends daycare or school**									
Yes	49.8 (42.2;57.3)	57.6 (42.8;71.1)	37.7 (28.0;48.5)	33.4 (27.5;39.7)	51.4 (35.5;66.9)	46.1 (40.5;51.9)	50.8 (42.8;58.7)	42.9 (38.7;47.2)	51.3 (44.6;58.0)
No	40.5 (32.5;49.0)	63.2 (56.2;69.6)	23.4 (10.2;45.0)	36.6 (28.4;45.5)	48.7 (29.9;67.8)	39.2 (31.8;47.2)	30.5 (20.4;42.9)	38.2 (33.2;43.4)	41.3 (32.1;51.3)
**Birth order**									
First	39.1 (29.9;49.0)	66.7 (54.8;76.8)	29.3 (19.5;41.5)	37.0 (30.2;44.3)	55.7 (33.1;76.2)	42.6 (36.0;49.4)	44.9 (32.4;58.1)	40.3 (35.7;45.0)	49.6 (39.2;60.0)
Second	54.8 (46.0;63.3)	53.0 (38.3;67.2)	37.8 (20.8;58.3)	28.5 (21.3;36.8)	42.5 (24.1;63.2)	48.6 (41.2;56.1)	43.3 (33.8;53.4)	43.6 (38.2;49.1)	44.6 (36.7;52.9)
Third or above	52.1 (42.4;61.7)	64.4 (48.9;77.3)	49.7 (37.4;62.1)	36.6 (26.6;47.8)	50.2 (39.5;60.9)	41.1 (31.7;51.2)	38.6 (20.6;60.3)	40.5 (33.8;47.4)	44.0 (28.8;60.4)
**Vaccinated exclusively in public services**									
Yes	51.3 (43.4;59.1)	64.0 (54.8;72.4)	55.2 (47.9;62.2)	39.9 (34.2;45.8)	60.2 (51.8;68.0)	50.8 (45.3;56.2)	55.1 (49.1;61.1)	47.7 (43.8;51.7)	57.5 (52.9;62.0)
No	30.1 (24.1;37.0)	53.4 (28.0;77.1)	18.8 (9.7;33.3)	22.9 (15.8;31.8)	22.0 (5.4;58.4)	22.8 (16.7;30.2)	21.6 (12.8;34.0)	23.3 (19.0;28.2)	24.7 (15.8;36.6)
**Difficulty in taking child to vaccination service**									
Yes	35.4 (22.1;51.3)	47.7 (26.3;70.1)	29.1 (17.7;43.8)	37.8 (24.1;53.8)	75.1 (54.6;88.3)	28.4 (10.4;57.4)	29.0 (13.9;50.8)	33.1 (21.2;47.6)	39.6 (24.2;57.4)
No	46.9 (40.5;53.4)	62.5 (54.5;70.0)	34.4 (22.9;48.1)	34.5 (29.6;39.8)	48.2 (32.1;64.6)	44.0 (39.0;49.1)	44.6 (37.1;52.4)	41.2 (37.7;44.9)	47.8 (41.4;54.2)
**Vaccination delay for any vaccine up to 6 months old**									
Yes	42.0 (34.2;50.2)	56.2 (46.6;65.3)	31.5 (20.0;45.8)	28.8 (22.8;35.6)	45.9 (31.6;61.0)	33.1 (26.6;40.3)	36.2 (29.7;43.3)	32.6 (28.3;37.2)	41.2 (35.1;47.5)
No	63.7 (57.3;69.7)	70.0 (54.9;81.8)	64.8 (56.3;72.4)	59.6 (49.5;68.9)	79.7 (67.2;88.3)	60.5 (54.4;66.3)	57.1 (42.6;70.5)	60.6 (55.9;65.2)	61.6 (49.6;72.3)
**Received on the same date the vaccines recommended at 4 months old**									
Yes	64.0 (58.4;69.3)	68.5 (58.2;77.3)	56.3 (49.8;62.7)	49.1 (42.6;55.6)	55.1 (38.2;70.9)	57.4 (51.2;63.3)	51.7 (41.9;61.3)	56.0 (51.5;60.4)	54.8 (47.0;62.4)
No	32.9 (22.8;44.9)	55.7 (31.4;77.6)	21.0 (6.8;49.1)	45.2 (33.1;57.8)	69.4 (59.1;78.2)	22.4 (13.8;34.1)	47.0 (32.9;61.6)	32.3 (25.5;40.0)	55.2 (44.4;65.5)
**Total**	46.2 (40.2;52.3)	61.6 (53.6;69.1)	34.4 (23.3;47.2)	34.6 (29.9;39.6)	50.3 (34.4;66.1)	44.2 (39.3;49.3)	43.3 (35.9;51.0)	41.3 (37.9;44.9)	47.1 (40.8;53.4)

Higher coverage was found in all the cities among children vaccinated exclusively in public services (p-value<0.05). There was no statistical difference in coverage among children whose parents or guardians reported difficulties in accessing vaccination services in almost all the cities, except in Petrópolis, where coverage was higher. (Table 3).

Delay in any vaccine scheduled up to 6 months old was associated with lower vaccination coverage in all capitals and interior region cities, with the exception of Sete Lagoas. Among children who received, on the same date, the vaccines recommended at 4 months old (second dose of DTcP-Hib-HepB, second dose of IPV, second dose of RV1 and second dose of PCV10), coverage was higher in Belo Horizonte, Vitória and São Paulo and in the capitals taken together (p-value<0.05) (Table 3). 

**Figure 1:**
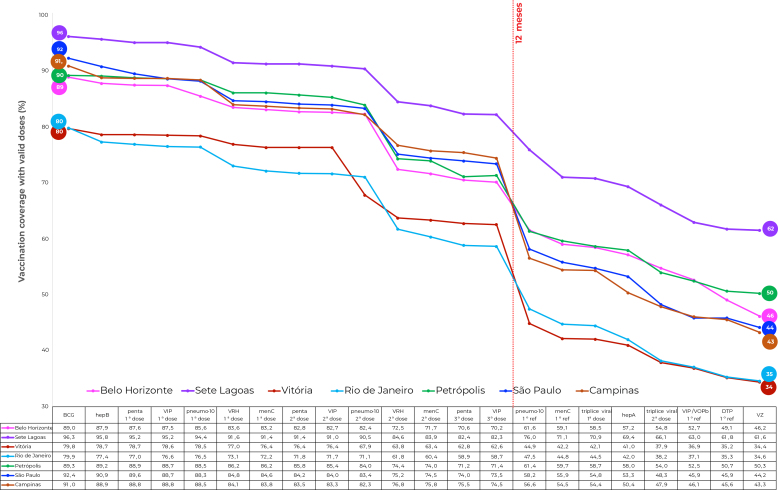
Coverage cascade showing valid doses of vaccines scheduled up to 24 months old, National Vaccination Coverage Survey, Belo Horizonte, Sete Lagoas, Vitória, Rio de Janeiro, Petrópolis, São Paulo and Campinas, Brazil, 2020 (n=8,703)

When the vaccine administration sequence is shown according to the PNI schedule (Figure 1), Sete Lagoas had the highest coverage, while Rio de Janeiro and Vitória had the lowest coverage over the entire sequence. The sequence in the cities was similar: the drops in coverage from the first to the last vaccine accounted for more than 40 percentage points, except in Sete Lagoas, and were more evident in the periods corresponding to administration of the second dose of RV1 and the first PCV10 booster.

The drop in coverage up to 12 months old was greatest in the city of Rio de Janeiro (21.2%) and lowest in Sete Lagoas (14.0%), with an average of 17.8% considering all the cities. Up to 15 months, drops in coverage were greatest in São Paulo (48.2%) and Campinas (47.7%), with an average of 43.3% considering all the cities (Figure 1). 

Taking the cities as a whole, the analysis of crude association (Table 4) showed lower odds of having full vaccination coverage with valid doses for children from families in socioeconomic strata A (OR=0.31; 95%CI 0.18;0.53), B (OR=0.32; 95%CI 0.21;0.48) and C (OR=0.65; 95%CI 0.48;0.87), in relation to those in stratum D; consumption levels A/B (OR=0.34; 95%CI 0.26;0.44), in relation to levels C/D; with monthly family income between BRL 3001 and BRL 8000 (OR=0.70; 95%CI 0.50;0.96) and greater than BRL 8000 (OR=0.23; 95%CI 0.14;0.39) compared to those who had income of less than BRL 1000; among those not benefitted by the federal income transfer program (OR=0.58; 95%CI 0.45;0.76; among children of mothers with higher education (OR=0.47; 95%CI 0.31;0.70), in relation to those with no schooling or with incomplete elementary education; among children vaccinated at least once in private services (OR=0.33; 95%CI 0.25;0.43); those who did not receive the recommended vaccines at 4 months of age on the same date (OR=0.41; 95%CI 0.28;0.59), and those who had any delayed vaccine up to 6 months old (OR=0 .32; 95%CI 0.25;0.41). 

**Table 4:** Crude and adjusted odds ratio (OR) between family, maternal and child characteristics and full coverage with valid doses, among children up to 24 months old, National Vaccination Coverage Survey, Belo Horizonte, Sete Lagoas, Vitória, Rio de Janeiro, Petrópolis, São Paulo and Campinas, Brazil, 2020 (n=8,703)

Variables	Crude OR (95%CI)	p-value	Adjusted OR (95%CI)	p-value
**Socioeconomic stratum**		<0.001		<0.001
A	0.31 (0.18;0.53)		0.39 (0.23;0.67)	
B	0.32 (0.21;0.48)		0.38 (0.25;0.58)	
C	0.65 (0.48;0.87)		0.75 (0.55;1.01)	
D	1.00		1.00	
**Family consumption level**		<0.001		<0.001
A/B	0.34 (0.26;0.44)		0.38 (0.28;0.52)	
C/D	1.00		1.00	
**Monthly family income (BRL)**		<0.001		<0.001
≤1000	1.00		1.00	
1001-3000	1.03 (0.75;1.42)		1.01 (0.73;1.40)	
3001-8000	0.70 (0.50;0.96)		0.69 (0.47;1.01)	
≥8001	0.23 (0.14;0.39)		0.23 (0.12;0.42)	
**Maternal schooling**		<0.001		<0.001
No schooling and incomplete elementary education	1.00		1.00	
Complete elementary education	1.27 (0.77;2.08)		1.25 (0.76;2.08)	
Complete high school education	1.15 (0.78;1.70)		1.15 (0.77;1.70)	
Complete higher education	0.47 (0.31;0.70)		0.47 (0.32;0.71)	
**Maternal age group (years)**		0.100		0.528
<20	1.00		1.00	
20-34	0.93 (0.37;2.31)		1.03 (0.36;2.98)	
35 or over	0.61 (0.25;1.50)		0.89 (0.29;2.75)	
**Income transfer program**				
Yes	1.00	<0.001	1.00	0.119
No	0.58 (0.45;0.76)		0.78 (0.57;1.07)	
**Maternal paid work**		<0.001		0.104
Yes	1.00		1.00	
No	1.48 (1.19;1.84)		1.21 (0.96;1.54)	
**Child vaccinated exclusively in public services**		<0.001		<0.001
Yes	1.00		1.00	
No	0.33 (0.25;0.43)		0.37 (0.26;0.51)	
**Child received on the same date the vaccines recommended at 4 months old**				<0.001
Yes	1.00	<0.001	1.00	
No	0.40 (0.28;0.59)		0.40 (0.27;0.59)	
**Child with vaccination delay for any vaccine up to 6 months old**		<0.001		<0.001
Yes	0.32 (0.25;0.41)		0.28 (0.22;0.37)	
No	1.00		1.00	

After adjustments, children from families in socioeconomic strata A and B remained less likely to have full vaccination coverage with valid doses (OR=0.39;95%CI 0.23;0.67 and OR=0.38; 95%CI 0.25;0.58); as did children from families with consumption levels A/B (OR=0.38; 95%CI 0.28;0.52); families with monthly income greater than BRL 8000 (OR=0.23; 95%CI 0.12;0.42); children who were not vaccinated exclusively in public services (OR=037; 95%CI 0.26;0.51); children who did not concomitantly receive the recommended vaccines at 4 months (OR=0.40; 95%CI 0.27;0.59); and children with any delay in vaccination up to 6 months old (OR=0.28; 95%CI 0.22;0.37) (Table 4).

## DISCUSSION

In this study, vaccination coverage of the full schedule with valid doses was below what is desirable to keep vaccine-preventable diseases under control in the four state capitals and three interior region cities in Southeast Brazil. This region of Brazil is made up of states with a tradition of producing and administering vaccines since the 19^th^ century.^14^

Trend analysis of vaccination coverage in 204 countries, between 1980 and 2019, using the third DTP dose as a marker, showed that in Brazil, coverage above 90%, found in the 1980s and 1990s, suffered drops of around 10% in the 2000s and more than 20% in the 2010s. This trend of poorer performance was found in Latin America, the Caribbean and the United States, in addition to Europe and Central Asia.^15^ Studies based on modeling that use coverage for each dose of vaccine do not allow coverage to be estimated for the full schedule, such as the coverage rates presented in this study, but can give an idea of global trends.

Despite the relative heterogeneity in coverage across the full scheme and the differences found in the longitudinal follow-up in each city, it was possible to identify a certain pattern for the seven cities studied in the Southeast region. This pattern is summarized as insufficient coverage, a continuous drop in coverage throughout the first year of life (less pronounced in the first half of the year and more intense in the second half of the year) and a significant drop in the second year of life. A similar pattern of progressive drops in coverage was found in a study conducted in a favela in the city of Recife, served by the Family Health Strategy, which found 84% full coverage for children under 6 months old, 68% for children under 12 months old and only 37% for those between 12 and 36 months old.^16^

In poorer countries such as Ethiopia, Pakistan, Nigeria and India, vaccination coverage inequalities are directly proportional to family wealth levels, generally reflecting different reasons for access difficulties.^17^ In Nepal, full schedule coverage fell between 2011 and 2016, while inequalities reduced, which is directly related to maternal schooling and wealth quintiles, which improved in the period.^18^

Low coverage was found in the cities in Southeast Brazil included in the national survey as a whole, but coverage was even lower when family socioeconomic status was higher (inverse relationship). This suggests reasons not directly related to access to vaccines that may be related to: greater use of private services (possibly with less adherence to the official schedule), difficulties in daily life and parental decisions. These reasons may favor certain vaccines or postpone administration of certain doses, creating their own vaccination schedule. This entire context is part of what is referred to as vaccination hesitancy.^19,20^

In a national survey carried out just over ten years ago with a cohort of children born alive in 2005, coordinated by the same group as the current survey, similar behavior was found in São Paulo, Rio de Janeiro and Belo Horizonte, but total coverage found on that occasion was above 70% and, in stratum A it was above 60%.^21^ Current data show significant worsening that may be related to the deepening of feelings of hesitation, failures associated with the performance of program actions in view of the defunding of the Brazilian National Health System, less importance being given to prevention and control programs in primary care centers and management of primary health care networks by social organizations without due regulation by Executive Branch bodies.^22^

The reductions in coverage also found in the other economic strata (B and C) were not found in the previous survey. This generalized worsening in coverage for the full schedule at 24 months may be related to the administrative problems already mentioned, in addition to the greater impoverishment of urban populations, causing access difficulties not directly related to the distribution of the primary care network, such as lack of money for transportation and mobility difficulties or, even, greater spread of vaccine hesitancy in parts of the population that had not previously been affected.

A study conducted in Araraquara, a city in the state of São Paulo with around 300,000 inhabitants, in the 2014-2016 cohorts, showed that coverage of administered doses was higher among income transfer program beneficiaries. This did not occur for on-time dose coverage, suggesting that income transfer program requirements have a limited effect on compliance with the schedule, when comparing only the poorest families.^23^

Our survey of cities in Southeast Brazil also indicated greater full vaccination coverage with valid doses among families that exclusively used public health services for vaccination and among children without vaccination delays in the first 6 months of life and who simultaneously received all scheduled vaccines in the fourth month of life. In the national survey, delay was one of the factors reducing coverage in the first two years of life. It is important to consider that delay may be related to parental decisions, difficulties in accessing health services, the complexity of the schedule or periods of vaccine scarcity, when supply is not regular, or lack of supplies or human resources in health services. The results from the seven cities in the Southeast did not show lower coverage for children whose mothers reported missed opportunities, that is, having taken the child to the health service without being able to get their child vaccinated.

A study carried out in 2016 in health services in the Philippines showed high proportions of delays (30 days or more) for the basic schedule (BCG, first DTP booster, bOPV, third HpeB dose, first dose of measles vaccine). Only 60.7% completed the basic schedule without delays. The factors associated with the occurrence of delays were the number of children (above 5) and lower maternal schooling.^24^

In Quebec, data from surveys carried out between 2008 and 2016 showed vaccination delays of 5.4%, 13.3%, 23.1% and 23.6% at 2, 4, 6 and 12 months, respectively. The authors concluded that 72.5% of incomplete schedules at 24 months could be attributed to delays in previous appointments.^25^

Several factors make it difficult to maintain high vaccination coverage rates, such as the increasing complexity of the vaccination schedule, defunding of the health sector, vaccination hesitancy, associated with faster vaccination reduction among children from high-income families,^26^ in addition to concerns about the safety of vaccines and religious and cultural beliefs.^27,28^ In contrast, perceptions about vaccine protection, recommendations made by health professionals, positive experiences in health services and favorable community attitudes facilitate adherence to vaccination.^27^ A combination of multiple strategies for different contexts and multiple barriers is necessary in order to improve vaccination coverage.^29^

Limitations of this study include data collection during the COVID-19 pandemic. The pandemic context impacted response rates, distributed asymmetrically between cities and population strata, which could affect the precision of the estimates, however, it is noteworthy that calculating post-stratification sampling weights minimized differences in responses between population groups. The inclusion of children only from urban areas makes it impossible to extrapolate the data to rural populations, small municipalities and minority groups. 

The main advantage of the survey is obtaining data directly from vaccination cards, with photographic records of the cards and data entry by experienced PNI professionals, which enables a longitudinal approach and calculation of coverage according to the full vaccination schedule.
