# Supplementary material for: Full vaccination coverage with valid doses among the 2017 and 2018 live birth cohort in the Southeast region of Brazil
Source: Epidemiol Serv Saude. 2024 Dec 16;33(spe2):e2024433. doi: 10.1590/S2237-96222024v33e2024433.especial2.en (PMC11654817; doi:10.1590/S2237-96222024v33e2024433.especial2.en)
Supplement: Translation

# Translation: Cobertura vacinal completa com doses válidas da coorte de nascidos vivos de 2017 e 2018 na região Sudeste do Brasil

**DOI:** 10.1590/S2237-96222024v33e2024433.especial2.pt

**Authors:** Ana Paula França, Rita Barradas Barata, Ione Aquemi Guibu, José Cássio de Moraes, Grupo ICV 2020, Adriana Ilha da Silva, Alberto Novaes Ramos, Ana Paula França, Andrea de Nazaré Marvão Oliveira, Antonio Fernando Boing, Carla Magda Allan Santos Domingues, Consuelo Silva de Oliveira, Ethel Leonor Noia Maciel, Ione Aquemi Guibu, Isabelle Ribeiro Barbosa Mirabal, Jaqueline Caracas Barbosa, Jaqueline Costa Lima, José Cássio de Moraes, Karin Regina Luhm, Karlla Antonieta Amorim Caetano, Luisa Helena de Oliveira Lima, Maria Bernadete de Cerqueira Antunes, Maria da Gloria Teixeira, Maria Denise de Castro Teixeira, Maria Fernanda de Sousa Oliveira Borges, Rejane Christine de Sousa Queiroz, Ricardo Queiroz Gurgel, Rita Barradas Barata, Roberta Nogueira Calandrini de Azevedo, Sandra Maria do Valle Leone de Oliveira, Sheila Araújo Teles, Silvana Granado Nogueira da Gama, Sotero Serrate Mengue, Taynãna César Simões, Valdir Nascimento, Wildo Navegantes de Araújo

**Affiliations:** 1 Faculdade de Ciências Médicas da Santa Casa de São Paulo, Departamento de Saúde Coletiva, São Paulo, SP, Brasil; Universidade Federal do Espírito Santo, Vitória, ES, Brasil; Universidade Federal do Ceará, Departamento de Saúde Comunitária, Fortaleza, CE, Brasil; Faculdade Ciências Médicas Santa Casa de São Paulo, São Paulo, SP, Brasil; Secretaria de Estado da Saúde do Amapá, Macapá, AP, Brasil; Universidade Federal de Santa Catarina, SC, Brasil; Organização Pan-Americana da Saúde, Brasília, DF, Brasil; Instituto Evandro Chagas, Belém, PA, Brasil; Faculdade de Ciências Médicas Santa Casa de São Paulo, Departamento de Saúde Coletiva, São Paulo, SP, Brasil; Universidade Federal de Mato Grosso, Cuiabá, MT, Brasil; Universidade Federal do Paraná, Curitiba, PR, Brasil; Universidade Federal de Goiás, Goiânia, GO, Brasil; Universidade Federal do Piauí, Teresina, PI, Brasil; Universidade de Pernambuco, Faculdade de Ciências Médicas, Recife, PE, Brasil; Instituto de Saúde Coletiva, Universidade Federal da Bahia, Salvador, BA, Brasil; Secretaria de Estado da Saúde de Alagoas, Maceió, AL, Brasil; Universidade Federal do Acre, Rio Branco, AC, Brasil; Universidade Federal do Maranhão, Departamento de Saúde Pública, São Luís, MA, Brasil; Universidade Federal de Sergipe, Aracaju, SE, Brasil; Secretaria Municipal de Saúde, Boa Vista, RR, Brasil; Fundação Oswaldo Cruz, Mato Grosso do Sul, Campo Grande, MS, Brasil; Fundação Oswaldo Cruz, Escola Nacional de Saúde Pública Sergio Arouca, Rio de Janeiro, RJ, Brasil; Universidade Federal do Rio Grande do Sul, Porto Alegre, RS, Brasil; Fundação Oswaldo Cruz, Instituto de Pesquisa René Rachou, Belo Horizonte, MG, Brasil; Secretaria de Desenvolvimento Ambiental de Rondônia, Porto Velho, RO, Brasil; Universidade de Brasília, Brasília, DF, Brasil

**Keywords:** Coberturas Vacinais, Saúde da Criança, Fatores Socioeconômicos, Inquéritos Epidemiológicos

## Abstract

**Objetivo:**

Analisar fatores associados à cobertura vacinal completa com doses válidas, em crianças das capitais e em três outras cidades da região sudeste.

**Métodos:**

Trata-se de análise de inquérito populacional conduzido em 2020-2021, em amostra estratificada segundo níveis socioeconômicos de crianças nascidas em 2017-2018, com dados coletados por registro fotográfico da caderneta de vacinação. Foram estimadas razões de chances (*odds ratio*, OR) e intervalo de confiança de 95% (IC95%) da cobertura vacinal completa pelas características da família, mãe e criança.

**Resultados:**

Em 8.703 crianças, menor cobertura ocorreu nos estratos A e B (OR=0,39; IC_95_% 0,23;0,67 e OR=0,38; IC_95_% 0,25;0,58); nível de consumo A/B (OR=0,38; IC_95_% 0,28;0,52); renda mensal inferior a R$ 8.000,00 (OR=0,23; IC_95_% 0,12;0,42); em filhos de mães com ensino superior (OR=0,47; IC_95_% 0,32;0,71); em crianças não vacinadas exclusivamente no serviço público (OR=0,37; IC_95_% 0,26;0,51) e com atraso vacinal até 6 meses (OR=0,28; IC_95_% 0,22;0,37).

**Conclusão:**

As coberturas não atingiram metas para controlar doenças imunopreveníveis e associaram-se negativamente ao maior nível socioeconômico.

## Contribuições do estudo

**Principais resultados:**

As coberturas vacinais para o esquema completo com doses válidas foram muito baixas, colocando em risco o controle das doenças imunopreveníveis nas quatro capitais e em mais três cidades do interior da região Sudeste do Brasil.

**Implicações para os serviços:**

Gestores e profissionais do Sistema Único de Saúde precisam conhecer os fatores associados às baixas coberturas vacinais e ao risco aumentado de abandono do esquema, os problemas de acesso e as dificuldades do Programa Nacional de Imunizações.

**Perspectivas:**

A compreensão dos determinantes das baixas coberturas vacinais dão subsídios para a discussão e o delineamento de políticas públicas eficazes para o enfrentamento às barreiras e a qualificação dos serviços de saúde para vacinação.

## INTRODUÇÃO

O Programa Nacional de Imunizações (PNI) instituído em 1973^1^ é reconhecido como um dos mais completos do mundo e por ter conseguido, durante anos, elevadas coberturas vacinais pelo caráter universal e gratuito do Sistema Único de Saúde (SUS).^2^

No entanto, a partir de 2012, detectou-se declínio das coberturas vacinais, acentuando-se em 2016 e, ainda mais, pela pandemia de covid-19.^3^ Dados do Sistema de Informação do Programa Nacional de Imunizações (SI-PNI) mostraram que, entre 2018 e 2019, o número de doses aplicadas de sete vacinas reduziu em todas as regiões brasileiras: rotavírus monovalente, pneumocócica 10 valente, hepatite B, BCG, poliomielite inativada e oral, difteria, tétano e pertussis e pentavalente. Apenas as coberturas das vacinas varicela e meningocócica conjugada aumentaram.^4^

Apesar do pioneirismo na implementação e no desenvolvimento de ações em imunização do PNI, o Sudeste, assim como as demais regiões, registrou queda nas coberturas vacinais, com patamares abaixo das metas do calendário nacional.^5^ Em 2020, as doses distribuídas em crianças até 10 anos caíram 9,4% em relação a 2019, com o Sudeste sendo uma das regiões mais afetadas (-12,8%).^6^ A cobertura contra a poliomielite, segundo dados do SI-PNI, diminuiu quase 30% entre 2011 e 2021, tendência observada em 24 dos 26 estados e no Distrito Federal.^7^

Considerando as médias das coberturas das vacinas do calendário infantil, disponibilizadas por unidade da federação no SI-PNI, foi constatado declínio preocupante no período de 2016 a 2020 em todos os estados do Sudeste: 24,0% no Espírito Santo, 22,5% em Minas Gerais, 50,3% no Rio de Janeiro e 28,1% em São Paulo.^8^

Cabe ressaltar que as coberturas vacinais obtidas com dados do SI-PNI e oriundas de inquéritos têm indicado discrepâncias, que sinalizam possíveis falhas no registro de doses aplicadas no sistema informatizado e na estimativa da população-alvo.^9^

Inquéritos domiciliares produzem estimativas mais precisas, permitindo estimar crianças com esquema vacinal completo e a proporção de suscetíveis, e compreender os determinantes socioeconômicos da distribuição heterogênea da cobertura vacinal infantil e fatores relacionados à equidade de acesso ao PNI.^10^

O objetivo deste artigo é analisar fatores associados à cobertura vacinal completa com doses válidas, em crianças das capitais e em três outras cidades da região sudeste.

## MÉTODOS

Trata-se de análise do Inquérito Nacional de Cobertura Vacinal, estudo de base populacional, conduzido de 2020 a 2022 em crianças nascidas em 2017 e 2018, residentes na área urbana das 26 capitais brasileiras, do Distrito Federal e em 12 cidades com mais de 100 mil habitantes. 

Neste estudo, verificou-se o cumprimento do calendário vacinal do PNI até os 24 meses de vida de crianças nascidas nas quatro capitais da região Sudeste (Belo Horizonte, MG; Vitória, ES; Rio de Janeiro, RJ e São Paulo, SP) e em três outras cidades da região (Sete Lagoas, MG; Petrópolis, RJ e Campinas, SP).

Na primeira etapa, classificaram-se os setores censitários em quatro estratos socioeconômicos (A, B, C e D, sendo o estrato A o de melhor condição socioeconômica, e o estrato D, o de pior) em cada um dos 39 municípios. Os pontos de corte de classificação dos estratos foram distintos entre os municípios, definidos segundo dados de renda nominal do chefe de família, percentual de chefes de família com renda superior a 20 salários mínimos e percentual de chefes de família alfabetizados, do Censo Demográfico 2010.^11^

O cálculo do tamanho amostral considerou cobertura vacinal de 70%, efeito de desenho de 1,4 e intervalo de confiança de 95%, o que resultou em 452 crianças em cada inquérito. O número de inquéritos em cada município foi definido com base na sua população, resultando em 452 crianças em Sete Lagoas e Petrópolis, 904 em Vitória e 1.808 crianças em Belo Horizonte, Rio de Janeiro, São Paulo e Campinas.^11^

Os setores foram agrupados por proximidade e número de crianças previsto, de modo que cada conglomerado contivesse três vezes o número de crianças a serem incluídas na amostra para compensar possíveis erros dos endereços e outras perdas. Os conglomerados foram sorteados de modo sistemático.^11^

As datas de aplicação das vacinas foram obtidas por registro fotográfico da caderneta de vacinação. As características socioeconômicas das famílias, mães e crianças e questões sobre hesitação vacinal foram obtidas por entrevista utilizando questionário estruturado aplicado por entrevistador treinado.

Foi realizada a junção, ou seja, a soma de doses de diferentes vacinas voltadas para a prevenção das mesmas doenças (por exemplo: tríplice viral [sarampo, caxumba e rubéola] e tetraviral), a fim de calcular corretamente as coberturas considerando as vacinas aplicadas pelo setor público e por serviços privados.^12^

A diferença entre as datas registradas na caderneta e a data de nascimento da criança permitiu classificar as doses como válidas, quando aplicadas desde 15 dias antes da data aprazada pelo PNI, respeitando o intervalo mínimo recomendado para cada dose.^12^

O desfecho foi a cobertura vacinal completa com doses válidas, definida pela aplicação de todas as doses e reforços das vacinas do calendário oficial do PNI, até os 24 meses: BCG; hepatite B (hepB); pentavalente (penta: DTP + *hemophilus influenza* B + hepatite B) – primeira + segunda + terceira doses; poliomielite 1, 2 e 3 inativada (VIP: primeira + segunda + terceira doses); rotavírus humano (VRH: primeira + segunda doses); meningocócica C (menC: primeira + segunda doses + reforço); pneumocócica 10-valente (pneumo 10: primeira + segunda doses); sarampo, caxumba e rubéola (tríplice viral: primeira + segunda doses + reforço); hepatite A (hepA: primeira dose); varicela (VZ: dose única); poliomielite 1 e 3 atenuada (VOPb: reforço); difteria, tétano e pertussis (DTP: reforço). A vacina febre amarela foi excluída do cálculo, pois não fazia parte do calendário de rotina em alguns municípios.^12^

As covariáveis incluíram os blocos a seguir.

- Características das famílias: estrato socioeconômico (A, B, C e D); nível de consumo familiar (A/B e C/D), definido segundo critério da Associação Brasileira de Empresas de Pesquisa;^13^ renda familiar mensal (≤R$ 1.000, R$ 1.001-R$ 3.000, R$ 3.001-R$ 8000, ≥R$ 8.001); aglomeração intradomiciliar (não, sim=≥3 pessoas por cômodo); e beneficiário do programa federal de transferência de renda (sim, não).
- Características maternas: escolaridade (sem instrução ou fundamental incompleto, fundamental completo ou médio incompleto, médio completo ou superior incompleto, superior completo); trabalho remunerado no último mês (sim, não); faixa etária (<20 anos, 20-34 anos, ≥35 anos); presença de companheiro (sim, não); e número de filhos (1, 2 e ≥3).
- Características das crianças: sexo (masculino, feminino); raça/cor da pele (branca, preta ou parda); frequência em creche e escola (sim, não); ordem de nascimento (primeiro, segundo, terceiro ou mais); dificuldade de acesso ao serviço de vacinação (sim, não); qualquer atraso vacinal até os 6 meses (sim, não); e se receberam na mesma data as vacinas recomendadas aos 4 meses (sim, não).

A cascata das coberturas para as vacinas previstas no calendário do PNI até os 24 meses iniciou com a BCG, calculada com o número de vacinados com doses válidas da referida vacina no numerador e, no denominador, o número de crianças que fizeram parte da amostra. Na sequência, o numerador foi o número de crianças que receberam doses válidas de determinada vacina e, o denominador, o número de doses válidas da vacina imediatamente anterior. Assim, para calcular a cobertura da hepB ao nascer, o número de crianças que receberam doses válidas dessa vacina foi dividido pelo número de crianças vacinadas com doses válidas da BCG.

As estimativas de cobertura vacinal e seus respectivos intervalos de confiança de 95% foram calculados no programa Stata®, versão 17, módulo *survey analysis*, considerando os pesos amostrais e o delineamento do estudo. Para testar as associações entre o desfecho e as covariáveis, utilizou-se o teste qui-quadrado de Pearson (p-valor<0,05). Por meio de regressão logística, foram estimadas razões de chances (*odds ratio*, OR) brutas e ajustadas por idade e escolaridade da mãe, com respectivos intervalos de confiança (IC_95_%). 

O estudo foi aprovado pelos comitês de ética em pesquisa com seres humanos do Instituto de Saúde Coletiva da Universidade Federal da Bahia, sob Parecer nº 3.366.818, em 4 de junho de 2019, com Certificado de Apresentação de Apreciação Ética (CAAE) 4306919.5.0000.5030; e da Irmandade da Santa Casa de São Paulo, sob Parecer nº 4.380.019, em 4 de novembro de 2020, com CAAE 39412020.0.0000.5479. 

## RESULTADOS

Fizeram parte deste estudo 8.703 crianças nascidas entre 2017 e 2018, em Belo Horizonte (n=1.863, 103,0% do previsto), Vitória (n=788, 87,2% do previsto), Rio de Janeiro (n=1.820, 100,7% do previsto), São Paulo (n=1.539, 85,1% do previsto), Sete Lagoas (n=451, 99,6% do previsto), Petrópolis (n=468, 103,5% do previsto) e Campinas (n=1.774, 98,1% do previsto).

Na região Sudeste, a proporção de crianças com cobertura vacinal completa com doses válidas aos 24 meses foi estimada em 41,3% (IC_95_% 37,9;44,9) para as capitais e em 47,1% (IC_95_% 40,8;53,4) para as três cidades do interior. A cidade com maior cobertura vacinal foi Sete Lagoas (61,6%; IC_95_% 53,6;69,1), e as com menores coberturas foram Vitória (34,4%; IC_95_% 23,3;47,2) e Rio de Janeiro (34,6%; IC_95_% 29,9;39,6). Nas demais cidades, as coberturas vacinais foram de 43,3% (IC_95_% 35,9;51,0) em Campinas, de 44,2% (IC_95_% 39,3;49,3) em São Paulo, de 46,2% (IC_95_% 40,2;52.3) em Belo Horizonte e de 50,3% (IC_95_% 34,4;66,1) em Petrópolis (Tabela 1). 

**Tabela 1:** Belo Horizonte, Sete Lagoas, Vitória, Rio de Janeiro, Petrópolis, São Paulo e Campinas, 2020 (n=8.703)

Características	**Minas Gerais**		Espírito Santo	**Rio de Janeiro**		São Paulo		Sudeste	
	**Belo Horizonte (n=1.863)**	**Sete Lagoas (n=451)**	**Vitória (n=788)**	**Rio de Janeiro (n=1.820)**	**Petrópolis (n=468)**	**São Paulo (n=1.539)**	**Campinas (n=1.774)**	**Capitais (n=6.010)**	**Interior (n=2.693)**
**Estrato socioeconômico**									
A	28,6 (21,3;37,1)	51,4 (38,2;64,4)	3,5 (0,9;12,4)	18,1 (6,5;41,5)	19,7 (4,5;55,9)	32,0 (19,0;48,5)	12,0 (5,7;23,6)	24,1 (15,2;35,9)	16,6 (9,3;28,0)
B	28,1 (19,4;39,0)	55,0 (42,6;66,9)	39,4 (28,5;51,5)	16,6 (8,9;28,8)	56,4 (47,4;65,1)	20,0 (11,3;32,8)	47,5 (37,2;58,0)	20,0 (14,0;27,8)	49,5 (41,2;57,8)
C	43,6 (35,3;52,2)	54,9 (41,9;67,3)	34,0 (27,4;41,3)	42,8 (34,9;51,1)	70,4 (53,7;83,0)	28,8 (20,7;38,5)	55,3 (49,0;61,5)	35,3 (29,5;41,7)	58,0 (51,8;64,0)
D	51,5 (41,9;61,0)	69,3 (55,7;80,2)	60,1 (51,9;67,7)	38,0 (31,8;44,6)	56,6 (48,3;64,7)	51,6 (45,8;57,3)	56,7 (49,4;63,8)	47,7 (43,4;52,1)	59,1 (53,6;64,5)
**Nível de consumo familiar**									
A/B	38,0 (28,6;48,4)	53,2 (33,8;71,7)	19,2 (10,1;33,3)	20,2 (13,7;28,9)	13,5 (3,4;40,5)	24,6 (18,5;31,9)	26,7 (16,7;39,8)	24,3 (20,0;29,3)	27,9 (18,5;39,7)
C/D	49,3 (41,4;57,3)	63,6 (54,5;71,9)	55,8 (48,9;62,5)	39,9 (34,0;46,1)	55,7 (43,6;67,2)	52,2 (46,6;57,7)	57,1 (52,3;61,7)	48,3 (44,3;52,4)	57,9 (53,5;62,1)
**Renda familiar mensal (R$)**									
≤1.000,00	57,5 (48,9;65,7)	52,3 (36,6;67,6)	64,5 (52,4;74,9)	38,6 (29,9;48,2)	45,3 (37,0;53,8)	53,5 (45,7;61,1)	55,0 (45,3;64,3)	49,8 (44,1;55,4)	52,1 (45,4;58,7)
1.001-3.000,00	49,9 (35,7;64,2)	66,2 (57,1;74,3)	52,8 (40,7;64,5)	41,7 (33,6;50,1)	55,3 (36,8;72,4)	53,8 (44,0;63,4)	56,1 (49,6;62,4)	50,0 (43,5;56,5)	57,6 (51,2;63,7)
3.001-8.000,00	41,4 (35,0;48,0)	69,0 (47,3;84,6)	30,7 (21,6;41,7)	40,6 (31,2;50,7)	46,1 (18,6;76,2)	39,8 (28,8;51,8)	46,4 (31,9;61,5)	40,1 (33,8;46,8)	48,1 (35,3;61,3)
≥8.001,00	23,6 (10,2;45,5)	30,2 (8,7;66,1)	19,1 (6,5;44,5)	21,0 (11,5;35,3)	10,1 (1,5;45,4)	14,1 (5,1;33,1)	18,3 (9,0;33,7)	18,6 (11,9;28,0)	18,6 (9,6;33,1)
**Aglomeração intradomiciliar**									
Sim	61,7 (41,8;78,4)	66,2 (33,1;88,6)	62,8 (45,5;77,3)	27,5 (15,9;43,4)	35,3 (22,9;50,1)	37,4 (25,6;51,0)	45,1 (31,7;59,2)	34,8 (26,0;44,7)	45,4 (34,5;56,8)
Não	45,5 (39,4;51,8)	61,4 (53,5;68,8)	32,9 (21,9;46,2)	36,2 (31,2;41,5)	51,3 (34,0;68,3)	45,1 (40,1;50,1)	43,2 (35,4;51,3)	42,3 (38,8;45,9)	47,2 (40,6;53,9)
**Programa de transferência de renda**									
Sim	51,6 (40,4;62,7)	56,2 (42,3;69,3)	65,9 (55,1;75,3)	49,7 (40,4;59,1)	44,6 (37,5;52,0)	53,5 (45,7;61,1)	49,7 (39,4;60,0)	52,5 (46,8;58,1)	49,6 (42,7;56,4)
Não	44,9 (38,3;51,6)	63,3 (55,4;70,5)	28,0 (17,8;41,2)	31,1 (25,9;36,8)	52,3 (30,9;72,8)	41,5 (35,9;47,4)	42,1 (33,5;51,3)	38,4 (34,6;42,5)	46,6 (39,0;54,4)
Total	46,2 (40,2;52,3)	61,6 (53,6;69,1)	34,4 (23,3;47,2)	34,6 (29,9;39,6)	50,3 (34,4;66,1)	44,2 (39,3;49,3)	43,3 (35,9;51,0)	41,3 (37,9;44,9)	47,1 (40,8;53,4)

Segundo características das famílias, as coberturas foram maiores no estrato socioeconômico D, em Belo Horizonte, Vitória, São Paulo e Campinas, e no estrato C, no Rio de Janeiro e em Petrópolis (p-valor<0,05). Em Sete Lagoas, não foi constatada diferença estatística segundo estratos. As coberturas vacinais menores nos estratos socioeconômicos mais altos (A e B) foram observadas na maior parte das cidades (p-valor<0,05), com exceção de Belo Horizonte e Sete Lagoas onde não houve diferença entre os estratos. A cobertura vacinal completa foi menor nas famílias com renda ≥R$ 8.001 na maior parte dos municípios (Belo Horizonte, Vitória, Rio de Janeiro, São Paulo e Campinas), assim como no conjunto das capitais e das cidades do interior (p-valor<0,05). Coberturas estatisticamente maiores foram observadas entre crianças de famílias beneficiárias do programa de transferência de renda de Vitória, do Rio de Janeiro, de São Paulo e no conjunto das capitais (Tabela 1).

A cobertura vacinal com doses válidas foi menor (p-valor<0,05) nos filhos de mães com maior escolaridade em Sete Lagoas, Vitória, São Paulo e Campinas e, também, no conjunto de capitais e cidades do interior. Filhos de mães com trabalho remunerado no mês que antecedeu a entrevista também tiveram cobertura vacinal com doses válidas menor (p-valor<0,05), com exceção de Sete Lagoas e Campinas. Filhos de mães com 35 anos ou mais também tiveram menores coberturas, em Vitória, São Paulo e no conjunto das capitais e cidades do interior (Tabela 2).

**Tabela 2:** Cobertura vacinal completa de doses válidas (%) e intervalo de confiança de 95% (IC95%) pelas características das mães, Inquérito Nacional de Cobertura Vacinal, Belo Horizonte, Sete Lagoas, Vitória, Rio de Janeiro, Petrópolis, São Paulo e Campinas, 2020 (n=8.703)

Características	**Minas Gerais**		Espírito Santo	**Rio de Janeiro**		São Paulo		Sudeste	
	**Belo Horizonte (n=1.863)**	**Sete Lagoas (n=451)**	**Vitória (n=788)**	**Rio de Janeiro (n=1.820)**	**Petrópolis (n=468)**	**São Paulo (n=1.539)**	**Campinas (n=1.774)**	**Capitais (n=6.010)**	**Interior (n=2.693)**
**Escolaridade**									
Sem instrução e fundamental incompleto	47,3 (31,9;63,2)	50,9 (29,5;72,0)	57,9 (39,8;74,0)	46,4 (30,0;63,7)	58,5 (36,3;77,7)	42,7 (33,1;53,0)	63,9 (46,0;78,6)	44,6 (36,4;53,2)	61,1 (48,3;72,5)
Fundamental completo	52,0 (43,4;60,5)	46,4 (28,7;65,0)	53,3 (41,4;64,9)	34,1 (22,9;47,3)	57,0 (45,0;68,3)	58,4 (46,6;69,4)	55,2 (43,2;66,6)	51,2 (42,5;59,9)	54,1 (46,0;61,9)
Médio completo	50,5 (39,1;61,9)	70,9 (63,5;77,3)	61,4 (52,1;69,9)	37,6 (29,7;46,2)	60,0 (48,6;70,4)	51,4 (44,7;58,1)	59,5 (53,4;65,4)	47,9 (42,9;52,9)	61,8 (57,0;66,4)
Superior completo	37,4 (30,3;45,0)	45,2 (30,8;60,4)	19,1 (10,5;32,4)	27,1 (20,9;34,2)	26,1 (7,0;62,6)	27,7 (21,3;35,1)	26,5 (17,2;38,3)	28,0 (23,8;32,8)	27,9 (19,2;38,7)
**Trabalho remunerado no último mês**									
Sim	41,2 (35,5;47,2)	63,7 (51,3;74,5)	26,6 (14,9;42,7)	30,4 (25,1;36,2)	42,0 (24,2;62,1)	40,7 (34,3;47,5)	42,1 (31,1;54,0)	37,4 (33,1;42,0)	44,4 (35,4;53,9)
Não	52,1 (43,1;60,9)	60,0 (49,3;69,8)	48,6 (41,0;56,2)	39,3 (32,6;46,5)	66,4 (52,2;78,1)	50,0 (43,4;56,5)	51,2 (40,1;62,1)	47,0 (42,4;51,6)	55,8 (47,7;63,6)
**Faixa etária (anos)**									
<20	50,6 (30,6;70,4)	76,7 (15,7;98,3)	38,8 (8,0;82,3)	44,6 (19,6;72,7)	35,6 (13,5;66,3)	48,8 (19,0;79,4)	91,2 (75,1;97,3)	47,6 (27,4;68,6)	78,0 (59,4;89,6)
20-34	46,3 (36,5;56,4)	61,9 (51,4;71,5)	48,7 (39,5;58,0)	38,0 (31,6;44,8)	60,4 (48,7;70,9)	50,4 (42,9;57,9)	51,1 (41,4;60,8)	46,1 (41,1;51,2)	54,8 (47,7;61,7)
≥35	46,0 (39,6;52,6)	61,1 (51,4;70,1)	25,0 (13,4;42,0)	30,8 (24,1;38,4)	36,4 (15,6;64,1)	38,2 (32,0;44,8)	36,8 (25,3;50,1)	36,4 (31,9;41,1)	39,8 (29,8;50,8)
**Companheiro**									
Sim	43,6 (36,0;51,4)	61,9 (52,7;70,3)	30,4 (19,2;44,5)	33,0 (27,9;38,5)	51,9 (30,9;72,2)	43,1 (37,1;49,2)	44,1 (35,5;53,0)	39,9 (35,8;44,2)	47,7 (40,2;55,4)
Não	51,4 (41,8;60,8)	61,2 (51,6;69,9)	50,9 (40,9;61,0)	37,7 (29,9;46,3)	44,4 (32,3;57,3)	49,5 (40,6;58,4)	52,3 (39,8;64,6)	45,9 (40,3;51,7)	52,3 (44,9;59,7)
**Número de filhos**									
1	42,6 (33,8;51,8)	63,8 (52,8;73,5)	27,4 (17,1;40,9)	38,6 (30,2;47,6)	59,3 (40,2;76,0)	43,6 (35,8;51,8)	46,5 (35,8;57,6)	41,6 (36,2;47,1)	51,2 (42,7;59,7)
2	49,5 (41,1;58,0)	60,0 (47,8;71,1)	36,5 (22,1;53,9)	28,8 (21,7;37,1)	43,6 (18,3;72,7)	45,2 (37,5;53,0)	43,5 (35,3;52,1)	40,8 (35,5;46,3)	45,7 (37,3;54,3)
≥3	46,9 (40,5;53,4)	60,2 (46,4;72,6)	51,5 (40,4;62,5)	35,6 (26,4;46,0)	50,9 (39,5;62,2)	43,9 (36,2;51,9)	37,5 (22,3;55,6)	41,7 (36,0;47,6)	42,9 (30,1;56,6)
**Total**	46,2 (40,2;52,3)	61,6 (53,6;69,1)	34,4 (23,3;47,2)	34,6 (29,9;39,6)	50,3 (34,4;66,1)	44,2 (39,3;49,3)	43,3 (35,9;51,0)	41,3 (37,9;44,9)	47,1 (40,8;53,4)

As características das crianças pouco interferiram nas coberturas vacinais com doses válidas nas cidades estudadas (Tabela 3). Não foram observadas diferenças estatisticamente significativas em relação ao sexo e à raça/cor da pele. A cobertura nas crianças que frequentavam escola e creche foi maior apenas em Campinas (p-valor<0,05). 

**Tabela 3:** Cobertura vacinal completa de doses válidas (%) e intervalo de confiança de 95% (IC95%) pelas características das crianças e barreiras dos serviços, Inquérito Nacional de Cobertura Vacinal, Belo Horizonte, Sete Lagoas, Vitória, Rio de Janeiro, Petrópolis, São Paulo e Campinas, 2020 (n=8.703)

Características	**Minas Gerais**		Espírito Santo	**Rio de Janeiro**		São Paulo		Sudeste	
	Belo Horizonte (n=1.863)	Sete Lagoas (n=451)	Vitória (n=788)	Rio de Janeiro (n=1.820)	Petrópolis (n=468)	São Paulo (n=1.539)	Campinas (n=1.774)	Capitais (n=6.010)	Interior (n=2.693)
**Sexo**									
Masculino	42,8 (34,2;52,0)	64,4 (53,6;74,0)	38,5 (26,0;52,6)	35,8 (29,5;42,6)	39,8 (21,9;61,0)	45,2 (39,0;51,6)	43,5 (34,2;53,2)	42,0 (37,7;46,4)	45,6 (37,7;53,8)
Feminino	50,5 (44,9;56,0)	58,4 (49,1;67,2)	29,4 (18,7;43,1)	33,3 (27,2;40,1)	66,1 (51,5;78,2)	43,2 (36,9;49,8)	43,1 (33,4;53,4)	40,7 (36,2;45,3)	48,8 (41,0;56,7)
**Raça/cor da pele**									
Branca	43,1 (36,0;50,5)	54,1 (39,5;68,1)	27,3 (18,7;37,9)	32,4 (26,4;39,1)	44,0 (23,6;66,6)	43,8 (38,2;49,6)	40,8 (30,7;51,7)	40,4 (36,2;44,7)	42,4 (33,6;51,6)
Preta/parda	49,2 (42,0;56,4)	63,4 (53,1;72,6)	42,9 (26,7;60,8)	38,6 (30,9;47,0)	59,5 (49,7;68,5)	45,8 (37,7;54,1)	49,8 (37,5;62,0)	43,7 (38,6;48,9)	55,0 (46,9;62,8)
**Frequenta creche ou escola**									
Sim	49,8 (42,2;57,3)	57,6 (42,8;71,1)	37,7 (28,0;48,5)	33,4 (27,5;39,7)	51,4 (35,5;66,9)	46,1 (40,5;51,9)	50,8 (42,8;58,7)	42,9 (38,7;47,2)	51,3 (44,6;58,0)
Não	40,5 (32,5;49,0)	63,2 (56,2;69,6)	23,4 (10,2;45,0)	36,6 (28,4;45,5)	48,7 (29,9;67,8)	39,2 (31,8;47,2)	30,5 (20,4;42,9)	38,2 (33,2;43,4)	41,3 (32,1;51,3)
**Ordem de nascimento**									
Primeiro	39,1 (29,9;49,0)	66,7 (54,8;76,8)	29,3 (19,5;41,5)	37,0 (30,2;44,3)	55,7 (33,1;76,2)	42,6 (36,0;49,4)	44,9 (32,4;58,1)	40,3 (35,7;45,0)	49,6 (39,2;60,0)
Segundo	54,8 (46,0;63,3)	53,0 (38,3;67,2)	37,8 (20,8;58,3)	28,5 (21,3;36,8)	42,5 (24,1;63,2)	48,6 (41,2;56,1)	43,3 (33,8;53,4)	43,6 (38,2;49,1)	44,6 (36,7;52,9)
Terceiro ou mais	52,1 (42,4;61,7)	64,4 (48,9;77,3)	49,7 (37,4;62,1)	36,6 (26,6;47,8)	50,2 (39,5;60,9)	41,1 (31,7;51,2)	38,6 (20,6;60,3)	40,5 (33,8;47,4)	44,0 (28,8;60,4)
**Vacinada exclusivamente no serviço público**									
Sim	51,3 (43,4;59,1)	64,0 (54,8;72,4)	55,2 (47,9;62,2)	39,9 (34,2;45,8)	60,2 (51,8;68,0)	50,8 (45,3;56,2)	55,1 (49,1;61,1)	47,7 (43,8;51,7)	57,5 (52,9;62,0)
Não	30,1 (24,1;37,0)	53,4 (28,0;77,1)	18,8 (9,7;33,3)	22,9 (15,8;31,8)	22,0 (5,4;58,4)	22,8 (16,7;30,2)	21,6 (12,8;34,0)	23,3 (19,0;28,2)	24,7 (15,8;36,6)
**Dificuldade para levar a criança o serviço de vacinação**									
Sim	35,4 (22,1;51,3)	47,7 (26,3;70,1)	29,1 (17,7;43,8)	37,8 (24,1;53,8)	75,1 (54,6;88,3)	28,4 (10,4;57,4)	29,0 (13,9;50,8)	33,1 (21,2;47,6)	39,6 (24,2;57,4)
Não	46,9 (40,5;53,4)	62,5 (54,5;70,0)	34,4 (22,9;48,1)	34,5 (29,6;39,8)	48,2 (32,1;64,6)	44,0 (39,0;49,1)	44,6 (37,1;52,4)	41,2 (37,7;44,9)	47,8 (41,4;54,2)
**Atraso vacinal em qualquer vacina até os 6 meses de vida**									
Sim	42,0 (34,2;50,2)	56,2 (46,6;65,3)	31,5 (20,0;45,8)	28,8 (22,8;35,6)	45,9 (31,6;61,0)	33,1 (26,6;40,3)	36,2 (29,7;43,3)	32,6 (28,3;37,2)	41,2 (35,1;47,5)
Não	63,7 (57,3;69,7)	70,0 (54,9;81,8)	64,8 (56,3;72,4)	59,6 (49,5;68,9)	79,7 (67,2;88,3)	60,5 (54,4;66,3)	57,1 (42,6;70,5)	60,6 (55,9;65,2)	61,6 (49,6;72,3)
**Recebeu na mesma data as vacinas recomendadas aos 4 meses de vida**									
Sim	64,0 (58,4;69,3)	68,5 (58,2;77,3)	56,3 (49,8;62,7)	49,1 (42,6;55,6)	55,1 (38,2;70,9)	57,4 (51,2;63,3)	51,7 (41,9;61,3)	56,0 (51,5;60,4)	54,8 (47,0;62,4)
Não	32,9 (22,8;44,9)	55,7 (31,4;77,6)	21,0 (6,8;49,1)	45,2 (33,1;57,8)	69,4 (59,1;78,2)	22,4 (13,8;34,1)	47,0 (32,9;61,6)	32,3 (25,5;40,0)	55,2 (44,4;65,5)
**Total**	46,2 (40,2;52,3)	61,6 (53,6;69,1)	34,4 (23,3;47,2)	34,6 (29,9;39,6)	50,3 (34,4;66,1)	44,2 (39,3;49,3)	43,3 (35,9;51,0)	41,3 (37,9;44,9)	47,1 (40,8;53,4)

Coberturas mais elevadas foram observadas entre as crianças vacinadas exclusivamente no serviço público em todas as cidades (p-valor<0,05). Não houve diferença estatística nas coberturas nas crianças cujos pais ou responsáveis referiram dificuldades de acesso ao serviço de vacinação em quase todos os municípios, exceto em Petrópolis, onde a cobertura foi maior (Tabela 3).

O atraso em qualquer vacina prevista até os 6 meses de idade associou-se a menores coberturas vacinais em todas as capitais e nas cidades do interior, com exceção de Sete Lagoas. Entre crianças que receberam, na mesma data, as vacinas preconizadas aos 4 meses de vida (segunda dose da penta, segunda dose da VIP, segunda dose da VRH e segunda dose da pneumo 10), as coberturas foram maiores em Belo Horizonte, Vitória e São Paulo e no conjunto das capitais (p-valor<0,05) (Tabela 3). 

Mostrando a sequência de aplicação das vacinas de acordo com o calendário do PNI (Figura 1), Sete Lagoas apresentou as coberturas mais elevadas, e Rio de Janeiro e Vitória apresentaram as menores em todo o trajeto. A evolução nas cidades foi parecida: as quedas de cobertura da primeira para a última vacina foram de mais de 40 pontos percentuais, exceto em Sete Lagoas, e foram mais evidentes nos períodos correspondentes à aplicação da segunda dose da VRH e no primeiro reforço da pneumo 10.

**Figura 1:** Cascata de cobertura de doses válidas das vacinas previstas até os 24 meses, Inquérito Nacional de Cobertura Vacinal, Belo Horizonte, Sete Lagoas, Vitória, Rio de Janeiro, Petrópolis, São Paulo e Campinas, 2020 (n=8.703)

A queda das coberturas até os 12 meses foi maior na cidade do Rio de Janeiro (21,2%) e menor em Sete Lagoas (14,0%), com média de 17,8% considerando todas as cidades. Até os 15 meses, as quedas foram maiores em São Paulo (48,2%) e Campinas (47,7%), com média de 43,3% considerando todas as cidades (Figura 1). 

A análise da associação bruta (Tabela 4) mostrou, para o conjunto de cidades do Sudeste, chances menores de ter cobertura vacinal completa com doses válidas as crianças de famílias dos estratos socioeconômicos A (OR=0,31; IC_95_% 0,18;0,53), B (OR=0,32; IC_95_% 0,21;0,48) e C (OR=0,65; IC_95_% 0,48;0,87), em relação às do estrato D; dos níveis de consumo A/B (OR=0,34; IC_95_% 0,26;0,44), em relação às dos níveis C/D; com renda familiar mensal entre R$ 3.001 e R$ 8.000 (OR=0,70; IC_95_% 0,50;0,96) e maior que R$ 8.000 (OR=0,23; IC_95_% 0,14;0,39) em comparação às que tinham renda inferior a R$ 1.000; em não beneficiárias do programa federal de transferência de renda (OR=0,58; IC_95_% 0,45;0,76; em filhos de mães com ensino superior (OR=0,47; IC_95_% 0,31;0,70), em relação às sem instrução ou com ensino fundamental incompleto; em crianças vacinadas ao menos uma vez em serviços privados (OR=0,33; IC_95_% 0,25;0,43); que não receberam na mesma data as vacinas preconizadas aos 4 meses (OR=0,41; IC_95_% 0,28;0,59), e as que tinham atraso em qualquer vacina até os 6 meses de vida (OR=0,32; IC_95_% 0,25;0,41). 

**Tabela 4:** Razão de chance (*odds ratio*, OR) bruta e ajustada entre as características das famílias, das mães e das crianças e a cobertura completa com doses válidas, em crianças com idade até 24 meses, Inquérito Nacional de Cobertura Vacinal, Belo Horizonte, Sete Lagoas, Vitória, Rio de Janeiro, Petrópolis, São Paulo e Campinas, 2020 (n=8.703)

Variáveis	OR bruta (IC_95_%)	*p*-valor	OR ajustada (IC_95_%)	*p*-valor
**Estrato socioeconômico**		<0,001		<0,001
A	0,31 (0,18;0,53)		0,39 (0,23;0,67)	
B	0,32 (0,21;0,48)		0,38 (0,25;0,58)	
C	0,65 (0,48;0,87)		0,75 (0,55;1,01)	
D	1,00		1,00	
**Nível de consumo familiar**		<0,001		<0,001
A/B	0,34 (0,26;0,44)		0,38 (0,28;0,52)	
C/D	1,00		1,00	
**Renda familiar mensal (R$)**		<0,001		<0,001
≤1.000,00	1,00		1,00	
1.001-3.000,00	1,03 (0,75;1,42)		1,01 (0,73;1,40)	
3.001-8.000,00	0,70 (0,50;0,96)		0,69 (0,47;1,01)	
≥8.001,00	0,23 (0,14;0,39)		0,23 (0,12;0,42)	
**Escolaridade da mãe**		<0,001		<0,001
Sem instrução e fundamental incompleto	1,00		1,00	
Fundamental completo	1,27 (0,77;2,08)		1,25 (0,76;2,08)	
Médio completo	1,15 (0,78;1,70)		1,15 (0,77;1,70)	
Superior completo	0,47 (0,31;0,70)		0,47 (0,32;0,71)	
**Faixa etária da mãe (anos)**		0,100		0,528
<20	1,00		1,00	
20-34	0,93 (0,37;2,31)		1,03 (0,36;2,98)	
35 ou mais	0,61 (0,25;1,50)		0,89 (0,29;2,75)	
**Programa de transferência de renda**				
Sim	1,00	<0,001	1,00	0,119
Não	0,58 (0,45;0,76)		0,78 (0,57;1,07)	
**Trabalho remunerado da mãe**		<0,001		0,104
Sim	1,00		1,00	
Não	1,48 (1,19;1,84)		1,21 (0,96;1,54)	
**Criança vacinada exclusivamente no serviço público**		<0,001		<0,001
Sim	1,00		1,00	
Não	0,33 (0,25;0,43)		0,37 (0,26;0,51)	
**Criança recebeu na mesma data as vacinas recomendadas aos 4 meses de vida**				<0,001
Sim	1,00	<0,001	1,00	
Não	0,40 (0,28;0,59)		0,40 (0,27;0,59)	
**Criança com atraso vacinal em qualquer vacina até os 6 meses de vida**		<0,001		<0,001
Sim	0,32 (0,25;0,41)		0,28 (0,22;0,37)	
Não	1,00		1,00	

Após ajustes, permaneceram com menor chance de ter cobertura vacinal completa com doses válidas as crianças das famílias dos estratos socioeconômicos A e B (OR=0,39; IC_95_% 0,23;0,67 e OR=0,38; IC_95_% 0,25;0,58); dos níveis de consumo A/B (OR=0,38; IC_95_% 0,28;0,52); com renda mensal superior a R$ 8.000 (OR=0,23; IC_95_% 0,12;0,42); crianças que não foram vacinadas exclusivamente em serviços públicos (OR=037; IC_95_% 0,26;0,51); que não receberam concomitantemente as vacinas preconizadas aos 4 meses (OR=0,40; IC_95_% 0,27;0,59); e com qualquer atraso vacinal até os 6 meses de vida (OR=0,28; IC_95_% 0,22;0,37) (Tabela 4).

## DISCUSSÃO

Neste estudo, as coberturas vacinais do esquema completo com doses válidas estiveram abaixo do desejável para manter as doenças imunopreveníveis sob controle nas capitais e em três cidades do interior do Sudeste brasileiro. Essa região é composta por estados com tradição na produção e aplicação de vacinas desde o século 19.^14^

Análise de tendência das coberturas vacinais em 204 países, entre 1980 e 2019, usando a terceira dose da DTP como marcador, mostrou que no Brasil, as coberturas acima de 90%, constatadas nas décadas de 1980 e 1990, sofreram quedas de cerca de 10% na década de 2000 e de mais de 20% na década de 2010. Essa tendência de pior desempenho foi verificada na América Latina, no Caribe e nos Estados Unidos, além da Europa e da Ásia Central.^15^ Estudos baseados em modelagens que usam coberturas para cada dose de vacina não permitem estimar as coberturas pelo esquema completo, como as apresentadas neste estudo, porém podem dar ideia das tendências mundiais.

Apesar da relativa heterogeneidade nas coberturas pelo esquema completo e das diferenças observadas no seguimento longitudinal em cada município, foi possível identificar certo padrão para os sete municípios estudados na região Sudeste. Esse padrão é resumido em coberturas insuficientes, queda contínua na cobertura ao longo do primeiro ano de vida (menos acentuada no primeiro semestre e mais intensa no segundo) e queda importante no segundo ano de vida. Padrão semelhante de quedas progressivas foi observado em estudo realizado em uma favela do Recife, atendida pela Estratégia Saúde da Família que constatou 84% de cobertura completa nos menores de 6 meses de idade, de 68% nos menores de 12 meses e apenas 37% entre 12 e 36 meses de idade.^16^

Nos países mais pobres como Etiópia, Paquistão, Nigeria e Índia, as desigualdades nas coberturas vacinais são diretamente proporcionais aos níveis de riqueza das famílias, geralmente refletindo diferentes motivos das dificuldades de acesso.^17^ No Nepal, as coberturas pelo esquema completo caíram entre 2011 e 2016, mas as desigualdades apresentaram redução, o que relaciona-se, diretamente à escolaridade das mães e aos quintis de riqueza, que melhoraram no período.^18^

Nas cidades do Sudeste do Brasil, incluídas no inquérito nacional, observaram-se coberturas baixas, mas ainda menores quanto melhores são as condições socioeconômicas das famílias (relação inversa). Isso sugere motivos não relacionados diretamente ao acesso das vacinas que podem estar relacionados com: o maior uso dos serviços privados (eventualmente com menor adesão ao calendário oficial), as dificuldades da vida cotidiana e as decisões dos pais. Estes podem privilegiar determinadas vacinas ou postergar a aplicação de certas doses, criando o próprio calendário de vacinação. Todo esse contexto faz parte da chamada hesitação vacinal.^19,20^

Em inquérito nacional realizado há pouco mais de 10 anos, coordenado pelo mesmo grupo do inquérito atual em uma coorte de nascidos vivos de 2005, observou-se comportamento semelhante em São Paulo, Rio de Janeiro e Belo Horizonte, porém as coberturas totais constatadas naquela ocasião estavam acima de 70% e, no estrato A, acima de 60%.^21^ Os dados atuais mostram piora relevante que pode estar relacionada ao aprofundamento dos sentimentos de hesitação, às falhas associadas ao desempenho das ações do programa tendo em vista o desfinanciamento do Sistema Único de Saúde, à menor importância dada aos programas de prevenção e controle nas unidades básicas e à gestão das redes básicas de saúde por meio de organizações sociais sem a devida regulação pelos órgãos do Poder Executivo.^22^

As reduções na cobertura observadas também nos outros estratos econômicos (B e C) não estavam presentes no inquérito anterior. Essa piora generalizada na cobertura para o esquema completo aos 24 meses pode estar relacionada aos problemas administrativos já mencionados, além do empobrecimento maior das populações urbanas, ocasionando dificuldades de acesso não relacionadas diretamente com a distribuição da rede de atenção básica, tais como a falta de dinheiro para locomoção e as dificuldades de mobilidade ou, ainda, maior penetração da hesitação vacinal em camadas da população que não tinham sido afetadas anteriormente.

Estudo realizado em Araraquara, cidade paulista com cerca de 300 mil habitantes, nas coortes de 2014 a 2016, mostrou que a cobertura é maior entre os beneficiários dos programas de transferência de renda para doses aplicadas. Isso não ocorreu para a cobertura com doses oportunas, sugerindo que as condicionalidades têm efeito limitado sobre o cumprimento do calendário, quando se comparam apenas as famílias mais pobres.^23^

O inquérito nas cidades do Sudeste também indicou maior cobertura vacinal completa com doses válidas nas famílias que utilizaram exclusivamente serviços públicos de saúde para vacinar e nas crianças sem atrasos nas vacinas nos primeiros 6 meses de idade e que receberam simultaneamente todas as vacinas previstas no quarto mês de idade. No inquérito nacional, o atraso foi um dos fatores de redução da cobertura nos dois primeiros anos de vida. É importante considerar que o atraso pode estar relacionado às decisões dos pais, às dificuldades de acesso aos serviços de saúde, à complexidade do calendário ou a períodos de escassez de vacinas, quando o abastecimento não é regular, ou à falta dos insumos ou recursos humanos nos serviços. Os resultados das sete cidades do Sudeste não mostraram menor cobertura para crianças cujas mães relataram oportunidades perdidas, isto é, ter levado a criança ao serviço sem conseguir que ela fosse vacinada.

Estudo realizado em 2016 em serviços de saúde nas Filipinas mostrou proporções altas de atrasos (30 dias ou mais) para o esquema básico (BCG, primeiro reforço da vacina DPT, VOPb, terceira dose da vacina contra hepB, primeira dose da vacina contra sarampo). Apenas 60,7% completaram o esquema básico sem atrasos. Os fatores associados à ocorrência de atrasos foram o número de filhos (acima de 5) e menor escolaridade materna.^24^

Em Quebec, dados de inquéritos realizados entre 2008 e 2016, mostraram atrasos vacinais de 5,4%, 13,3%, 23,1% e 23,6% aos 2,4,6 e 12 meses, respectivamente. Os autores concluíram que 72,5% dos esquemas incompletos aos 24 meses podiam ser atribuídos aos atrasos ocorridos em visitas anteriores.^25^

Diversos fatores que dificultam a manutenção das altas taxas de cobertura vacinal, como a complexidade crescente do calendário vacinal, o desfinanciamento do setor saúde, a hesitação em vacinar, associada ao declínio mais rápido em crianças de famílias de renda alta,^26^ além de preocupações com a segurança das vacinas e crenças religiosas e culturais.^27,28^ Em contraposição, percepções sobre a proteção das vacinas, recomendações de profissionais de saúde, experiências positivas nos serviços de saúde e atitudes favoráveis da comunidade facilitam a adesão.^27^ A combinação de múltiplas estratégias para os diferentes contextos e múltiplas barreiras são necessárias para melhoria da cobertura vacinal.^29^

Diversos fatores dificultaram a manutenção das altas taxas de coberturas vacinais, como o contexto pandêmico que impactou as taxas de resposta, distribuídas de modo assimétrico entre as cidades e os estratos populacionais, que poderiam afetar a precisão das estimativas, porém, destaca-se que o cálculo de pesos amostrais pós-estratificação minimizou diferenças nas respostas entre grupos populacionais. A inclusão apenas de crianças de áreas urbanas impossibilita a extrapolação de dados para populações rurais, de municípios de pequeno porte e grupos minoritários. 

A principal vantagem do inquérito está na obtenção dos dados diretamente nas cadernetas de vacinação, com registro fotográfica das cadernetas e digitação dos dados por profissionais experientes no PNI, o que possibilita a abordagem longitudinal e o cálculo das coberturas pelo esquema vacinal completo.
